# Probable Simulation of Isolated Right Ventricular Myocardial Infarction by Anterior Myocardial Infarction

**Published:** 2019-07

**Authors:** Ali Hosseinsabe, Mohammad Alidoosti

**Affiliations:** 1 Associate Professor of Cardiology, Department of Cardiology, Tehran Heart Center, Tehran University of Medical Sciences, North Karegar Street, Tehran, Iran. 1411713138. Tel: +98 21 88029731. Fax: +98 21 88029731. E-mail: ali_hosseinsabet@yahoo.com.; 2 Associate Professor of Cardiology, Department of Cardiology, Tehran Heart Center, Tehran University of Medical Sciences, North Kargar Street, Tehran, Iran. 1411713138. Tel: +98 21 88029256. Fax: +98 21 88029256. E-mail: salidoosti@hotmail.com.

A 38-year-old man with a history of cigarette smoking and hypertension presented to our emergency department with atypical chest pain. He had a history of non–ST-elevation myocardial infarction 2 weeks earlier, for which he had been admitted to another center and treated medically. Electrocardiography showed an ST elevation in lead V_1_ and nonspecific ST-T changes in the other limbs and the left precordial leads (Figure 1). Right precordial electrocardiography showed an ST-segment elevation in leads V_2_R to V_6_R (Figure 2). Accordingly, he was referred to the catheterization laboratory for primary percutaneous coronary intervention. Selective coronary angiography showed 100% stenosis in the mid-portion of the left anterior descending artery, which was treated via coronary stenting (Video 1). The other coronary arteries had insignificant stenosis. The right ventricular branch was normal (Video 2). Transthoracic echocardiography showed mesocardia (Figure 3 & Video 3) without other congenital defects. The left ventricular ejection fraction was about 45%, alongside hypokinesia in the mid-anteroseptal, mid-inferoseptal, and apicoseptal portions. The right ventricular systolic function was intact. Chest X-ray was in favor of mesocardia (Figure 4). It appeared that the rotation of the base-to-apex axis from left to midline might have resulted in the probable simulation of an isolated right ventricular myocardial infarction by an anterior myocardial infarction. Therefore, an anterior myocardial infarction in the presence of mesocardia should be considered in the differential diagnosis of an isolated right ventricular infarction.

**Figure 1:**
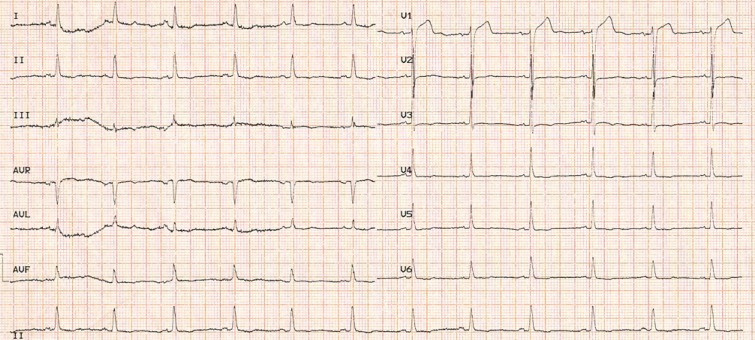
Admission time electrocardiography (limbs and left precordial leads), demonstrating an ST elevation in lead V_1 _and nonspecific ST-T changes in the other limbs and the left precordial leads

**Figure 2:**
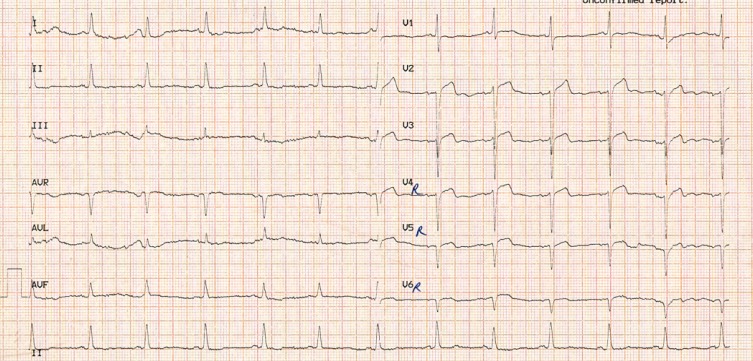
Admission time electrocardiography (limbs and right precordial leads), demonstrating an ST elevation in leads V_2_R to V_4_R

**Figure 3:**
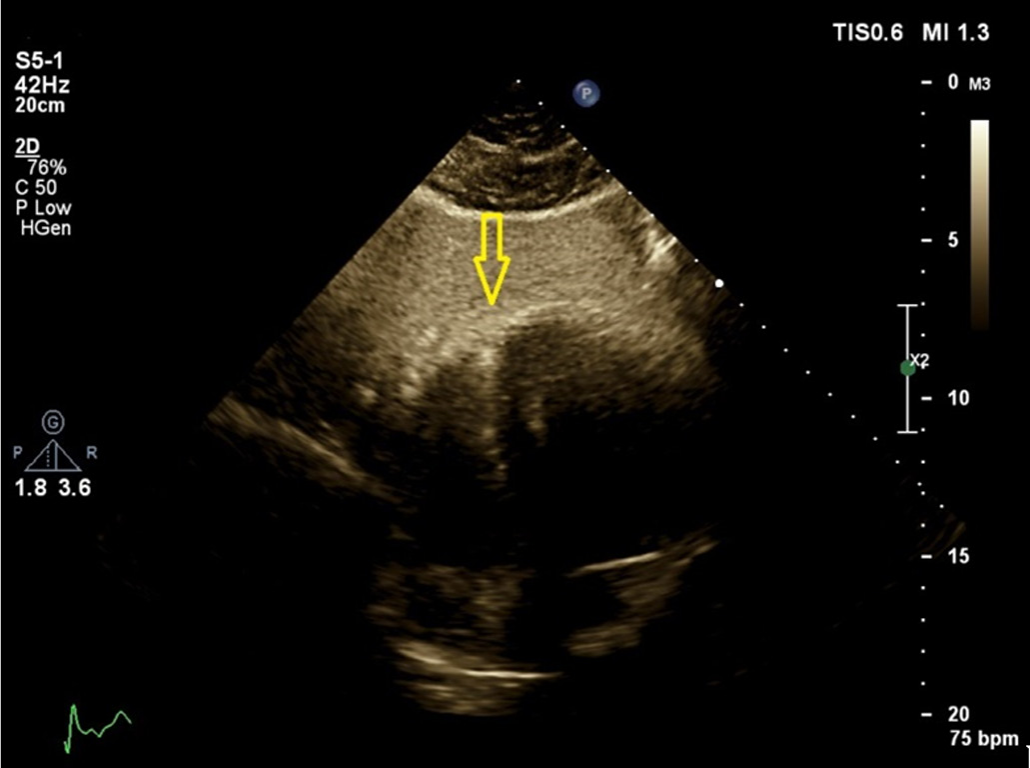
Mesocardia in the subcostal view in transthoracic echocardiography. The arrow shows the cardiac apex, which is in the midline and is suggestive of mesocardia.

**Figure 4:**
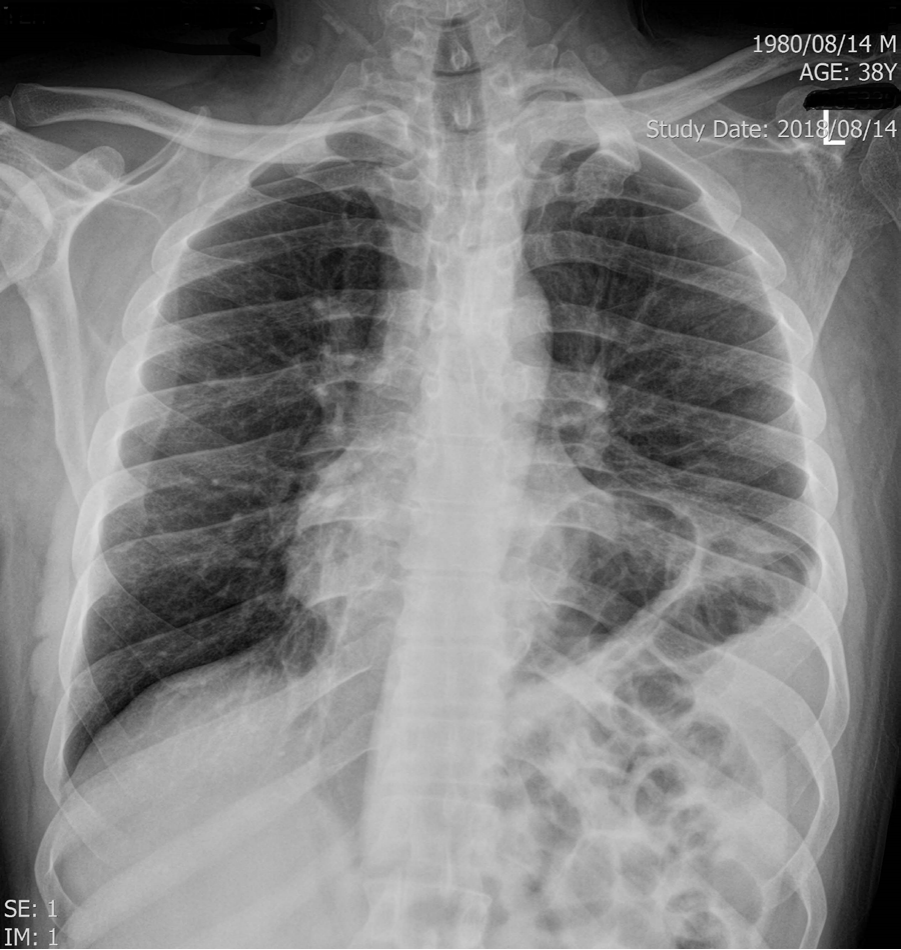
Chest X-ray in the posterior-anterior view, showing that the cardiac apex is not on the left or the right

## Notes:

***To watch the following videos, please refer to the relevant URLs: ***

http://jthc.tums.ac.ir/index.php/jthc/article/view/987/858

Videos 1. Selective coronary angiography, demonstrating 100% stenosis in the mid-portion of the left anterior descending artery

http://jthc.tums.ac.ir/index.php/jthc/article/view/987/859

Video 2. Selective coronary angiography, demonstrating insignificant stenosis in the right coronary artery and normal right ventricular branches

http://jthc.tums.ac.ir/index.php/jthc/article/view/987/860

Video 3. Transthoracic echocardiography, showing mesocardia in the subcostal view

